# Deep Learning Predicts EBV Status in Gastric Cancer Based on Spatial Patterns of Lymphocyte Infiltration

**DOI:** 10.3390/cancers13236002

**Published:** 2021-11-29

**Authors:** Baoyi Zhang, Kevin Yao, Min Xu, Jia Wu, Chao Cheng

**Affiliations:** 1 Department of Chemical and Biomolecular Engineering, Rice University, Houston, TX 77030, USA; bz26@rice.edu; 2 Department of Electrical and Computer Engineering, Texas A&M University, College Station, TX 77843, USA; ky0148@tamu.edu; 3 Computational Biology Department, Carnegie Mellon University, Pittsburgh, PA 15213, USA; mxu1@cs.cmu.edu; 4 Computer Vision Department, Mohamed bin Zayed University of Artificial Intelligence, Abu Dhabi 144534, United Arab Emirates; 5 Department of Imaging Physics, Division of Diagnostic Imaging, The University of Texas MD Anderson Cancer Center, Houston, TX 77030, USA; jwu11@mdanderson.org; 6 Department of Medicine, Baylor College of Medicine, Houston, TX 77030, USA; 7 Dan L. Duncan Comprehensive Cancer Center, Baylor College of Medicine, Houston, TX 77030, USA; 8 Institute for Clinical and Translational Research, Baylor College of Medicine, Houston, TX 77030, USA

**Keywords:** deep learning, gastric cancer, EBV, lymphocyte, prognosis

## Abstract

**Simple Summary:**

EBV infection represents a distinct subtype in gastric cancer, so determining infection status is important in guiding treatment decisions. Currently, EBV infection in gastric cancer is most often determined using PCR and in situ hybridization, which requires multiple steps and nucleic acid preservation. On the other hand, histopathology images are widely available and included in the course of diagnosis for patients. Thus, our development of an approach to determine EBV status from these histopathology images could save costs and time associated with making EBV diagnoses for gastric cancer patients or independently validate the results from traditional methods. Additionally, our model’s predictions are able to classify patients into EBV infection categories that are significantly correlated with prognosis. This may serve to better inform clinicians’ decisions in prescribing immunotherapy, as both EBV infection status and prognosis are critical factors in whether immunotherapy is effective or worth the costs and side effects.

**Abstract:**

EBV infection occurs in around 10% of gastric cancer cases and represents a distinct subtype, characterized by a unique mutation profile, hypermethylation, and overexpression of PD-L1. Moreover, EBV positive gastric cancer tends to have higher immune infiltration and a better prognosis. EBV infection status in gastric cancer is most commonly determined using PCR and in situ hybridization, but such a method requires good nucleic acid preservation. Detection of EBV status with histopathology images may complement PCR and in situ hybridization as a first step of EBV infection assessment. Here, we developed a deep learning-based algorithm to directly predict EBV infection in gastric cancer from H&E stained histopathology slides. Our model can not only predict EBV infection in gastric cancers from tumor regions but also from normal regions with potential changes induced by adjacent EBV+ regions within each H&E slide. Furthermore, in cohorts with zero EBV abundances, a significant difference of immune infiltration between high and low EBV score samples was observed, consistent with the immune infiltration difference observed between EBV positive and negative samples. Therefore, we hypothesized that our model’s prediction of EBV infection is partially driven by the spatial information of immune cell composition, which was supported by mostly positive local correlations between the EBV score and immune infiltration in both tumor and normal regions across all H&E slides. Finally, EBV scores calculated from our model were found to be significantly associated with prognosis. This framework can be readily applied to develop interpretable models for prediction of virus infection across cancers.

## 1. Introduction

Gastric cancer is the third leading cause of cancer-related death worldwide [1]. Epstein–Barr Virus (EBV) infection has been implicated in gastric cancer development. Around 10% of gastric cancers are associated with EBV infection, which represents a distinct subtype [2]. When compared with EBV negative gastric cancers, EBV positive gastric cancers display distinctive genetic and epigenetic tumorigenic profiles. EBV positive gastric cancers exhibit a distinct somatic mutation pattern, including prevalent PIK3CA mutations and rare TP53 mutations [3,4]. Epigenetically, EBV positive gastric cancers are characterized by intensive DNA hypermethylation [5]. In addition, high expression levels of programmed death ligands 1 (PD-L1) and PD-L2 are observed among EBV positive gastric cancers [3,6].

Histologically, EBV positive gastric cancers appear to be poorly differentiated, with abundant immune infiltration in their tumor microenvironment in comparison to EBV negative cancers [3,7]. Indeed, specific immune cells such as B and T lymphocytes are significantly higher in EBV positive gastric cancers and are associated with better overall survival rates [8]. With high expressions of immune checkpoint proteins such as PD-L1, EBV positive gastric cancers are considered to be promising candidates for immunotherapy. Past studies show that EBV positive patients have good response rates to anti-PD-1 therapy [9], as well as a longer progression free survival time after anti-PD-1 therapy [10]. Additionally, targeting therapies against VEGF receptors have been performed for EBV positive patients, and some of them have shown promising results [11]. Taken together, these results suggest great clinical importance for detecting EBV positive gastric cancers, as well as exploring immune infiltration in conjunction with EBV for a more complete picture of the tumor microenvironment. 

EBV positive gastric cancer was initially detected by the presentation of EBV DNA through polymerase chain reaction [12]. To date, in situ hybridization of EBV-encoded small RNA is the standard method for detecting EBV infection in gastric cancer samples [13]. Both of these methods cannot provide EBV status results in a single diagnostic step and require well-preserved nucleic acids in samples. Meanwhile, deep learning has shown great promise in analyzing medical images, including H&E histology slides, which can be obtained right through diagnosis. Recent studies have demonstrated that deep learning can accurately detect genetic variants, copy number alteration, and molecular subtypes in H&E slides [14,15]. A recent study even utilized deep learning to accurately predict HPV infection in oropharyngeal squamous cell carcinoma with an AUC around 0.8 [16]. 

In this study, we developed a deep learning-based algorithm that predicts EBV infection status from H&E slides. We explored the efficacy of our model in predicting EBV infection on tumor regions and on normal regions in each H&E slide. Interestingly, we observed the heterogeneity of our model’s predictions on different regions of the same whole-slide image. In addition, we investigated the correlation of immune infiltration levels with the predicted EBV score, specifically in patient cohorts with low and even zero EBV abundance, which led us to hypothesize that our model’s prediction is at least partially driven by spatial immune cell composition. This hypothesis was tested by exploring the local spatial correlations between the EBV score and immune infiltration per tile in the whole slide. Finally, we examined the prognostic utility of EBV scores and observed favorable prognosis in high score patients. 

## 2. Materials and Methods

### 2.1. Patient Cohorts

Diagnostic slides of gastric cancer patients from The Cancer Genome Atlas (TCGA) project were downloaded from the Genomic Data Commons Portal (https://portal.gdc.cancer.gov/ (accessed on 15 March 2021)). EBV abundance of these patients, represented by numbers of virus-supporting reads per hundred million reads processed (RPHM) from RNA-seq data, were obtained from a previous publication [17]. Taken together, 400 diagnostic slides from 375 patients were included in this study. Out of the 375 patients, 241 patients have EBV abundance information available. Specifically, out of the 241 patients, 24 patients with EBV abundance over 100 were considered as EBV+, while 98 patients with both zero EBV abundance and HHV5 abundance were considered as EBV−. EBV− patients were required to have zero HHV5, a virus from the same family as EBV, to prevent potentially confounding HHV5 effects. The remaining 119 patients formed a leave-out set that is characterized by low but non-zero EBV or zero EBV but non-zero HHV5 (Figure S1). This leave-out set was considered as EBV status unclear and was not used to train or test the EBV prediction model. 

### 2.2. Image Preprocessing

All 400 diagnostic slides were in SVS format at either 20× or 40× magnification. Slides with a magnification of 40× were first resized to 20×. Then, these slides were tessellated into square tiles of 512 × 512 pixels, approximately 250 × 250 µm, using OpenSlide [18]. Tiles with insufficient length or width at slide boundaries were dismissed. Additionally, tiles with more than 50% background, defined as RGB values over 220, were disregarded. To avoid potential impacts of stain intensity variance across H&E slides, all tiles were later color-normalized using the Macenko method [19]. 

### 2.3. Model Training

Our model architecture was based on Resnet18 [20], with the last layer modified. After modification, the last layer contained two or three nodes, each representing one of the three classes (tumor, dense normal and loss normal) or one of the two classes (EBV+ and EBV−). Pre-trained weights on the ImageNet database were downloaded. As a first step, we trained a model to distinguish tumor and normal tiles from TCGA gastric cancer slides. Specifically, this tumor vs. normal model was trained on an annotated dataset of 11,977 tiles from gastrointestinal cancer patients [21]. In this dataset, each tile was annotated according to 3 categories: tumor, dense and loose normal tissue. The tiles were randomly divided into training, validation, and test sets at a ratio of 0.7:0.15:0.15. Image augmentations including random reflection and translation within 5 pixels horizontally and vertically were applied to these tiles before feeding them into the model with a batch size of 64. Adam optimizer was used to update weights under learning rate of 5 × 10^−6^ and L2-regularization of 1 × 10^−^^5^. When loss on the validation set stops decreasing for 10 epochs, we stop training and evaluate the model on the test set. We achieved an overall accuracy at 0.9989, with per class accuracy at 0.9983 for each category (Figure S2), where per class accuracy was calculated as percentage of correctly predicted samples in a specific class and overall accuracy was calculated as percentage of correctly predicted samples in all classes.

Using the tumor vs. normal model, all tiles from the TCGA gastric cancer cohort were then divided into tumor and normal tiles. Specifically, normal tiles are those predicted to be either dense or loose normal tissues. Tumor tiles from EBV+ and EBV− samples, as previously described, were first divided into training and external test sets in a ratio of 0.7:0.3 on sample level (patient level). According to patient’s EBV status, the EBV status of each tile was assigned. To address imbalance problems, random downsampling of EBV− tiles was performed to match EBV+ tiles’ sample size in the training set. All tiles in the training set were further divided into training, validation and internal test sets with a ratio of 0.85:0.125:0.025 on the tile level (Figure S1). For simplicity, “external test set” is henceforth referred to as “test set”, unless otherwise stated. With a batch size of 256, the same image augmentation strategies were applied before feeding the tiles into the Resnet18 model [20]. Similarly, we used Adam optimizer to update weights under learning rate of 1 × 10^−6^ and L2-regularization of 5 × 10^−4^. In addition, to prevent overfitting, only weights after the last block, including the last block, were trained instead of the full model as before. When loss on the validation set stops decreasing for 5 epochs, we stop training. Since adjacent normal tissues can receive signals from tumor tissues and exhibit altered pathways [22], the same training procedure was performed on normal tiles to examine if our model can also capture potential changes in normal tissues induced by EBV+ tumor tissues. All model training and tuning procedures were implemented using python with PyTorch framework [23].

For EBV models trained on either tumor (tEBV model) or normal (nEBV model) tiles, a sample-level EBV score is calculated by the proportion of predicted EBV+ tiles across all tumor or normal tiles from each sample. Area under the curve (AUC) of the receiver operating characteristic was used to evaluate model performance on test sets. Specifically, slides with too few normal tiles (fewer than 10) or with handwriting on normal regions were considered as low quality and discarded for evaluation and downstream analysis of the nEBV model. 

### 2.4. Sampl-Level Immune Infiltration

The lymphocyte levels for each sample were estimated by analysis of corresponding H&E slides from a previous study [24]. An algorithm called BASE, which uses rank-based methods to examine the expression of immune cell-specific genes, was applied to calculate immune infiltration scores for specific immune cell subtypes [25]. Corresponding expression profiles, level 3 processed TCGA RNA-seq data for gastric cancer, were downloaded from FireBrowse (http://firebrowse.org (accessed on 15 March 2021)). Six major immune cell subtypes were focused, including Memory B cells, naïve B cells, CD4+ T cells, CD8+ T cells, NK cells and monocytes. 

### 2.5. Tile-Level Immune Infiltration

Tumor-infiltrating lymphocyte (TIL) maps for TCGA gastric cancer patients were downloaded from a previous publication through The Cancer Imaging Archive (TCIA) portal [26,27,28]. These TIL maps were also calculated from H&E slides. On these maps, lymphocyte infiltration was indicated by the color red. Tile position information was mapped to TIL maps. Each tile corresponds to a square of about 5 × 5 pixels or a square of about 2.5 × 2.5 pixels depending on the scale of TIL maps. Because pixels are not divisible, in practice, we calculated the pixel coordinates of a tile’s four vertices and rounded them to integers. Tile-level immune infiltration was then represented by the proportion of red pixels among all pixels belonging to the tile. 

### 2.6. Statistical Analysis

Wilcoxon test was used to quantify tile- and sample-level EBV score or immune infiltration difference between 2 groups. Gene Ontology enrichment analysis of gene sets was performed with function “enrichGO” from R package “clusterProfiler” [29]. Pearson correlation was used to quantify local correlations between immune infiltration and EBV score per slide. Specifically, tiles from some slides correspond to about 2.5 × 2.5 pixels in TIL maps, rounding edge length to 2 or 3 pixels will result in large errors in estimation of tile-level immune infiltrations. Therefore, these slides, as well as slides with insufficient normal tiles or handwriting, were dismissed for correlation tests on normal regions. 

### 2.7. Survival Analysis

Progression free survival times were obtained from a previous publication [24]. R packages “survival” and “survminer” were used in the survival analysis. Specifically, R functions “survfit” and “ggsurvplot” were used to generate Kaplan–Meier plots. The difference between survival curves was compared through the “survdiff” function. Corresponding *p* values were calculated through log-rank tests. Univariate and multivariate Cox regression models were built for EBV scores with “coxph” function. Confounding variables including lymphocyte level, age, sex and tumor stage were adjusted with multivariate Cox regression model. Specifically, we considered patients over 65 years old as old, stage I–II as low stage and III–IV as high stage.

## 3. Results

### 3.1. A Deep-Learning-Based Framework for Classifying EBV+ versus EBV− Gastric Tumors

**Figure 1:**
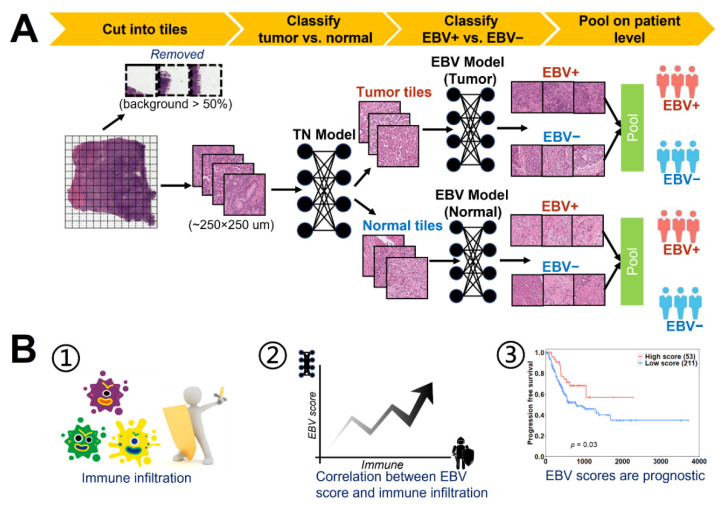
Schematic design of the study: (**A**) Deep-learning-based pipeline for predicting EBV status. The tumor vs. normal model (TN model) was first applied to distinguish tumor and normal tiles, and EBV models were then trained on tumor and normal tiles separately to predict EBV status. (**B**) Downstream analysis of models predicting EBV scores. (1) Comparing immune infiltration between samples with high and low EBV scores led us to hypothesize that our model was at least partially influenced by regional immune infiltration. (2) To test this hypothesis, we examined local correlations between EBV scores and immune infiltration. In spite of high correlations, our model outperformed predictions based solely on immune infiltrations. (3) Besides, our model can provide additional prognostic values compared to EBV status and immune infiltration.

We developed a convolutional neural network architecture based on Resnet18 [20] to classify EBV+ and EBV− gastric tumors. To start with, a tumor vs. normal model was first trained to detect tumor tiles from H&E slides. Then, an EBV model was trained on tumor tiles with 250 × 250 µm and generated EBV scores for each tile. A sample-level EBV score was calculated by dividing the number of predicted EBV+ tiles (EBV score > 0.5) by the sample’s total tumor tiles. Since adjacent normal tissues can receive signals from tumor tissues and exhibit altered pathways [22], a model was also trained on normal tiles in the same way (Figure 1A). The AUC from the sample-level EBV score was used to evaluate model performance. 

Since previous studies reported significant high immune infiltration in EBV+ samples [7], we extend the comparison of immune infiltration to cohorts with low EBV abundance (0~100 RPHM) and to cohorts with zero EBV abundance. Considering the similar significant differences observed, we hypothesized that the EBV model prediction was at least partially driven by spatial immune cell composition. To test this hypothesis, the correlation between the immune infiltration and EBV scores of the tiles in each slide were examined. In addition, we evaluated the prognostic utility of EBV models and found that higher EBV scores predict better survival (Figure 1B).

### 3.2. The EBV Model Accurately Predicts EBV+ Status

**Figure 2:**
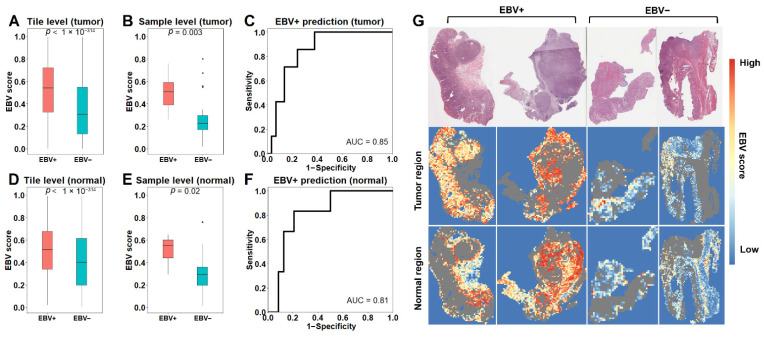
Performance of the model for predicting EBV status: (**A**,**B**) EBV scores calculated by the model applied to tumor tiles (tEBV model) distinguish EBV+ from EBV− on both tile level (**A**) and sample level (**B**). (**C**) Receiver Operating Characteristic (ROC) for classifying EBV+ and EBV− samples based on tEBV scores. (**D**,**E**) EBV scores derived from normal tiles (nEBV scores) distinguish EBV+ from EBV− on tile (**D**) and sample level (**E**). (**F**) Receiver Operating Characteristic (ROC) for classifying EBV+ and EBV− samples based on nEBV scores. (**G**) The first row shows 2 EBV+ and 2 EBV− gastric cancer H&E slides. The model prediction in tumor and normal regions is shown in the second and third rows, with grey indicating remaining tissues. *p* values are calculated from two-sided Wilcoxon rank-sum test.

Having trained the EBV model on tumor tiles (tEBV model), we then applied it to a test set of samples it had never seen before. As shown, tiles from EBV+ samples had significantly higher EBV scores than those from EBV− samples (*p* < 1 × 10^−314^, Figure 2A). We next calculated a sample-level EBV score as the proportion of tiles with EBV score over 0.5 out of total tumor tiles. Consistently, EBV+ samples also had significantly higher EBV scores than EBV− samples (*p* = 0.003, Figure 2B). To evaluate model performance, we applied the sample-level EBV scores to discriminate EBV+ versus EBV− samples and achieved an AUC of 0.85 (Figure 2C). Since adjacent normal tissues can receive signals from tumor tissues and exhibit altered pathways [22], we further trained the EBV model on normal tiles (nEBV model) to examine whether EBV infection in tumor tissues can induce changes within adjacent normal tissues that can be captured by our model. Similarly, normal tiles from EBV+ samples showed significant higher EBV scores to those from EBV− samples (*p* < 1 × 10^−314^, Figure 2D). As a result, EBV+ samples scored higher than EBV− samples (*p* = 0.02, Figure 2E), resulting in an AUC of 0.81 for distinguishing EBV+ samples (Figure 2F). We next applied the two EBV models to the leave-out set, which had samples with low EBV abundance. Specifically, samples with an EBV score over 0.5 were considered as high score samples, while the rest were low score samples. Using tEBV model, we found that high score samples were present in 20 of the 119 (17%) leave-out samples, consistent with 4 of the 29 (14%) EBV− samples in the test set (Figure S3A). Similar results were observed using the nEBV model, with 17 high scores in 103 (17%) leave-out samples and 3 high scores in 24 (12%) EBV− samples in the test set (Figure S3B). Taken together, Figure S3C showed the EBV scores from both models across the whole cohort. Most samples fell in the lower left and upper right, suggesting a high consistency between the tEBV model and the nEBV model. Some samples fell in the upper left and lower right, indicating inconsistent predictions between the two models for these samples. Figure 2G visualizes model performance in both tumor and normal regions for two EBV+ and two EBV− representative slides. As shown, tiles from EBV+ slides tend to have higher EBV scores compared to those from EBV− slides.

### 3.3. Association of EBV Status with Immunological Features

**Figure 3:**
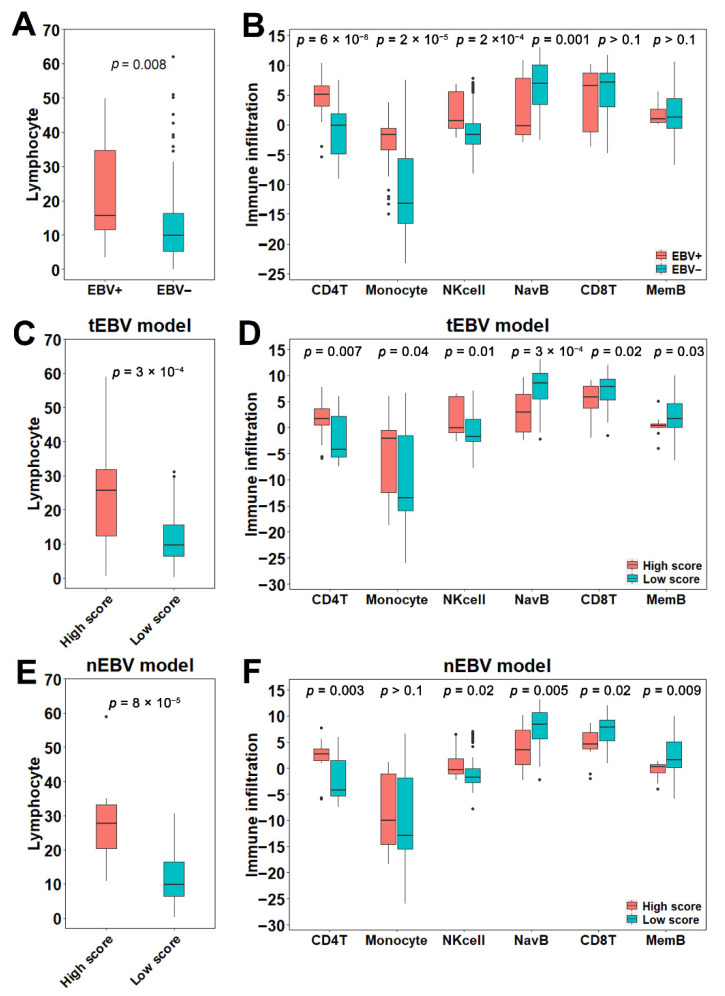
Association of EBV status with immune infiltration: (**A**) Differential lymphocyte infiltrations between EBV+ and EBV− samples. (**B**) Differential infiltrations of CD4+ T cells, monocytes, Natural killer cells and naïve B cells between EBV+ and EBV− samples. (**C**,**D**) Infiltration of lymphocytes (**C**) and six major immune subtypes (**D**) in tumor regions for high and low tEBV score samples with low EBV abundance. (**E**,**F**) Infiltration of lymphocytes (**E**) and six major immune subtypes (**F**) in normal regions for high and low nEBV score samples with low EBV abundance. tEBV or nEBV scores above 0.5 are considered as high scores. *p* values are calculated from one-sided (**A**,**C**,**E**) or two-sided (**B**,**D**,**F**) Wilcoxon rank-sum test.

Previous studies reported high immune infiltrations in EBV+ samples [7], which was confirmed in our analysis by the higher levels of lymphocytes in 24 EBV+ samples compared to 98 EBV− samples (*p* = 0.008, Figure 3A). The lymphocyte levels were estimated at the sample level by analysis of corresponding H&E slides from a previous study [24]. Then, we examined infiltration of specific immune cell subtypes between EBV+ and EBV− samples. Using a previously developed algorithm [25] based on expression data, we determined the levels of six major immune cell subtypes: naïve B cells, memory B cells, CD4+ T cells, CD8+ T cells, Natural killer cells and monocytes. We found that the infiltrations of CD4+ T cells, monocytes and Natural killer cells were significantly higher in EBV+ samples, while the infiltration of naïve B cells was significantly higher in EBV− samples (Figure 3B). 

Next, we extended our analysis to comparisons of high and low score samples in cohorts with low or zero EBV abundance. Specifically, since these cohorts from the leave-out set contained samples with zero EBV but some HHV5, a virus homolog of EBV, we first examined potential impacts of HHV5 on immune infiltration and EBV scores. To avoid EBV infection’s impact, only samples with zero EBV abundance from the entire cohort were kept for this analysis. We observed low and non-significant correlation coefficients between HHV5 abundance and lymphocyte infiltration (R = −0.096, Figure S4A), indicating that HHV5 does not significantly affect lymphocyte infiltration. Furthermore, HHV5 abundance was poorly correlated with the EBV scores from both the tEBV model (R = −0.018, Figure S4B) and the nEBV model (R = 0.042, Figure S4C). Based on this result, we extracted 86 samples with low (<100 RPHM) but non-zero EBV abundance regardless of HHV5 in the leave-out set and applied the tEBV and nEBV models on them to compare immune infiltration between high and low score samples. We considered EBV scores above 0.5 as high scores for both the tEBV model and the nEBV model. Using the tEBV model, 16 high score and 70 low score samples were identified. High score samples were found to have higher lymphocyte infiltrations with a more significant *p* value (*p* = 3 × 10^−4^, Figure 3C), which is expected because EBV+ has higher lymphocyte infiltration than EBV-. Consistently, high infiltration of CD4+ T cell, monocyte, and Natural killer cell were observed in high score samples, whereas low infiltration of naïve B cells was observed (Figure 3D). A significant increase in CD8 T cell and memory B cells in low score samples was also observed, even though their infiltration was comparable between EBV+ and EBV− samples. Similarly, using the nEBV model, an even more significant higher lymphocyte level was observed in 12 high score samples compared to 61 low score samples, after excluding samples with low quality normal tiles (*p* = 8 × 10^−5^, Figure 3E). Consistent with the tEBV model, significant differences in the six major immune cell subtypes, except for monocytes, between high and low score samples were validated (Figure 3F). Although not significant, the same trend was observed for monocytes. 

**Figure 4:**
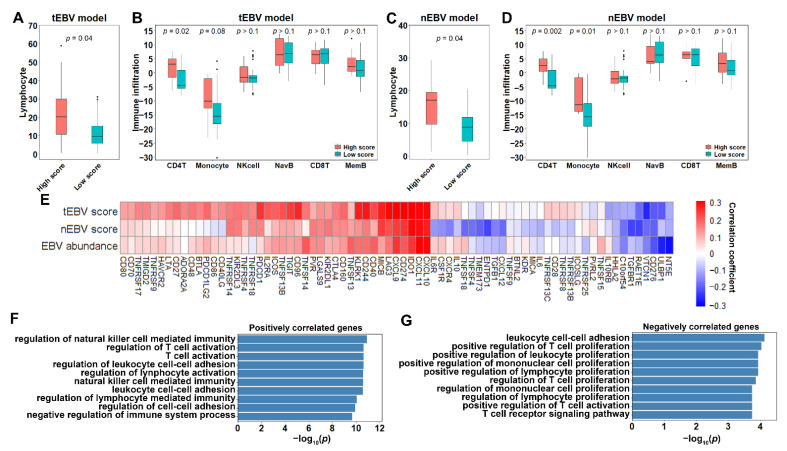
Association of EBV scores with immune infiltration: (**A**,**B**) Infiltration of lymphocyte (**A**) and six major immune cell subtypes (**B**) in tumor regions for high and low tEBV score samples with zero EBV abundance. (**C**,**D**) Infiltration of lymphocyte (**C**) and six major immune cell subtypes (**D**) in normal regions for high and low nEBV score samples with zero EBV abundance. (**E**) Correlation of expression of immune-related genes with tEBV score, nEBV score and EBV abundance over the entire cohort. (**F**,**G**) Enriched gene ontologies for positively (**F**) and negatively (**G**) correlated genes. *p* values are calculated from one-sided (**A**,**C**) or two-sided (**B**,**D**) Wilcoxon rank-sum test.

Having shown immune infiltration difference between high and low score samples with low (but not zero) EBV abundance, we then compared immune infiltration difference between high and low score samples with zero EBV abundance. Specifically, samples from the training set were excluded because training forced the model itself to fit towards correct EBV status, rather than immune infiltration. We utilized 29 EBV− samples from the test set, but because of the small sample size, and since HHV5 did not affect immune infiltration nor EBV score, we also combined 33 samples with zero EBV abundance from the leave-out set with the test set. We first applied tEBV model, resulting in 8 high score and 54 low score samples. As expected, the high score samples were highly infiltrated by lymphocytes compared to the low score samples (*p* = 0.04, Figure 4A). In terms of the six immune cell subtypes, CD4 T cells were found to be present at high levels in high score samples as in our previous results (Figure 4B). The same trend was observed for monocytes, but it did not reach significance threshold. Similarly, after excluding samples with low quality normal tiles, we applied our nEBV model and identified 8 high and 45 low score samples. Consistently, high score samples showed high levels of lymphocytes, as well as CD4 T cells and monocytes (Figure 4C,D). 

In addition, we examined the correlations of 73 immune-related genes to EBV score and EBV abundance within the entire cohort. This gene list included 70 major immunostimulatory and suppressor genes, as well as 3 genes (CXCL9, CXCL10, CXCL11) [3] known to be overexpressed in EBV+ that are used as positive controls. As shown in Figure 4E, the majority of genes (51 out of 73) showed positive correlations with EBV abundance. When both EBV models were applied, similar results were observed, with 53 out of 73 and 38 out of 73 genes correlated positively with EBV scores from the tEBV model and the nEBV model, respectively. Specifically, immune checkpoint genes CD274 and IDO1, as well as CXCL9, CXCL10 and CXCL11, were strongly positively correlated with EBV score and abundance. To better understand the role these genes played in the difference between EBV+ and EBV−, we performed Gene Ontology enrichment analyses of both positively and negatively correlated gene sets. In practice, we considered genes with correlation coefficients above 0.1 for tEBV score, nEBV score, and EBV abundance as positively correlated genes and those below −0.1 as negatively correlated genes. In total, 19 positively and 6 negatively correlated genes were identified. As shown, positively correlated genes were enriched on biological processes related to immune cell activation (Figure 4F), while negatively correlated genes were enriched on biological processes related to immune cell proliferation (Figure 4G). We further expanded the 73 immune-related genes to include all available 20,501 genes. To avoid too-large gene sets, we changed the threshold from ±0.1 to ±0.2 and identified 379 positively and 843 negatively correlated genes. Positively correlated genes were found to be enriched for interferon-gamma-related processes (Figure S5A), while negatively correlated genes were found to be enriched for development and molecular transport processes (Figure S5B).

### 3.4. The EBV Model Prediction Is Partially Driven by Regional Immune Infiltration in H&E Slides

**Figure 5:**
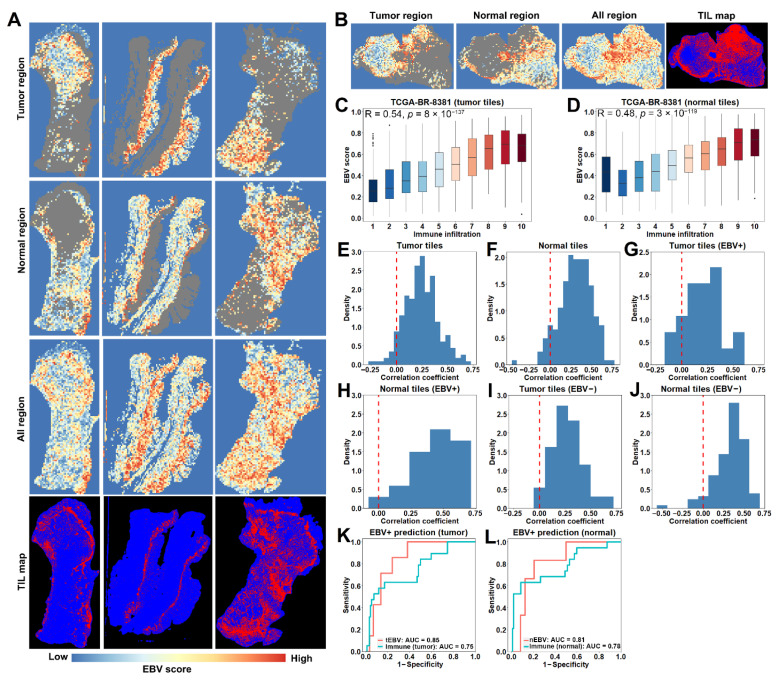
Local correlation between EBV scores and regional immune infiltration: (**A**) EBV score heatmaps match well with tumor-infiltrating lymphocyte (TIL) maps. (**B**–**D**) An example (**B**) shows high correlations of regional immune infiltration with EBV scores in both tumor (**C**) and normal (**D**) regions. (**E**,**F**) Majority of slides have positive correlations of regional immune infiltration with EBV scores in tumor (**E**) or normal (**F**) regions. (**G**–**J**) Stratifying slides by EBV abundance results in predominant positive correlations in slides with EBV+ (>100 RPHM) (**G**,**H**) and EBV− (0 RPHM) (**I**,**J**) abundance. (**K**,**L**) EBV models outperform predictions based on immune infiltration in tumor (**K**) or normal (**L**) regions.

Having shown different immune infiltration between high and low score samples in cohorts of patients with low or zero EBV abundance, we hypothesized that regional immune infiltration in H&E slides was contributing to the EBV model prediction. To test this hypothesis, we first visually compared model prediction results with tumor-infiltrating lymphocyte (TIL) maps, which were derived from a trained deep learning algorithm to distinguish lymphocyte-infiltrated patches based on a previous study [26,27,28]. As shown in Figure 5A, the heat maps of the EBV scores in both normal and tumor regions from some slides were found to be matched extremely well with the corresponding TIL maps. Taking a slide as an example (Figure 5B), we further quantified the correlation of regional immune infiltration with EBV scores in both tumor and normal regions. Specifically, we mapped tiles’ positions to the corresponding TIL map and calculated tile-level immune infiltration, represented by lymphocyte-infiltrated area divided by tile area. Furthermore, tile-level immune infiltrations were categorized into 10 levels, corresponding to the ratio of lymphocyte pixels to total number of pixels in the area: level 1 would be tiles with a ratio of 0~0.1, level 2 would be 0.1~0.2, and up to level 10, which would be 0.9~1. As shown, tiles with higher immune infiltrations had higher EBV scores, resulting in high correlation coefficients in both tumor (R = 0.54, Figure 5C) and normal regions (R = 0.48, Figure 5D). We then extended our analyses to all H&E slides. After excluding low quality slides, we found high correlations between immune infiltration and EBV scores from both tumor and normal regions across slides, with positive correlations in 213 out of 227 tumor regions (Figure 5E) and in 182 out of 197 normal regions (Figure 5F). According to EBV abundance, we next stratified the H&E slides into 3 categories: 25 EBV+ (>100 RPHM), 116 EBV− (0 RPHM) and 86 low EBV abundance (0~100 RPHM). Among EBV+ slides, 21 out of 25 and 21 out of 21 slides were observed with positive correlations for tumor (Figure 5G) and normal regions (Figure 5H), respectively. These results were further validated in EBV− slides, with 113 out of 116 slides and 94 out of 101 slides exhibiting positive correlations in tumor (Figure 5I) and normal regions (Figure 5J). Similar results were found for slides with low EBV abundance: 79 out of 86 and 67 out of 75 slides had positive correlations in their tumor (Figure S6A) and normal regions (Figure S6B). 

Since the EBV score is highly correlated with immune infiltration, we next examined whether immune infiltration in tumor or normal regions could predict EBV status. Specifically, given that HHV5 do not affect immune infiltration nor EBV score, we considered samples with zero EBV abundance as EBV− here regardless of HHV5, resulting in 24 EBV+ samples and 131 EBV− samples. We first calculated immune infiltration in the tumor region for each sample based on the lymphocyte-infiltrated area in the tumor region divided by the total tumor area according to TIL maps. As a result, we achieved an AUC of 0.75 with immune infiltration in tumor regions, lower than that from the tEBV model (AUC = 0.85, Figure 5K). Similar results were observed for immune infiltrations in normal regions, indicated by an AUC of 0.78 versus 0.81 from the nEBV model (Figure 5L). Taken together, the prediction ability of the EBV model is at least partially but not fully explained by immune infiltrations.

### 3.5. EBV Score Is Associated with Patient Prognosis

**Figure 6:**
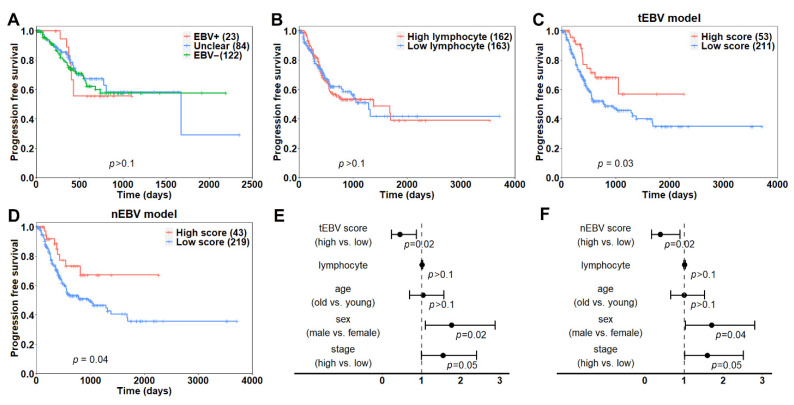
Association of EBV scores with patient prognosis: (**A**) Kaplan–Meier plots for EBV+ (>100 RPHM), unclear (0~100 RPHM) and EBV− (0 RPHM) patient groups. (**B**) Survival difference between high and low lymphocyte-infiltrating groups. The two groups were divided according to median lymphocyte levels. (**C**,**D**) Survival difference between high and low EBV score patients using tEBV (**C**) and nEBV (**D**) models. EBV scores above 0.5 were considered as high scores. (**E**,**F**) Forest plot for visualizing prognostic utilities of tEBV (**E**) and nEBV (**F**) scores from the multivariate Cox regression model.

Having shown that our model is partially driven by regional immune infiltrations, we next examined whether our model could provide additional prognostic value beyond immune infiltration and EBV status. To start with, we compared survival differences among patients with EBV+ (>100 RPHM), unclear (0~100 RPHM) and EBV− (0 RPHM) status. As shown in Figure 6A, all three groups of patients showed comparable progression-free survival rates. Next, we examined how immune infiltration affects progression free survival. After dichotomizing the patients into high and low lymphocyte-infiltrating groups based on their median value, we observed no significant survival differences between the two groups (Figure 6B). Additionally, we calculated EBV scores for each sample by applying the EBV models. After excluding training samples, patients with high scores were found to have significantly longer progression-free survival compared to those with low scores using both the tEBV (*p* = 0.03, Figure 6C) and nEBV (*p* = 0.04, Figure 6D) models. After considering established clinical variables including lymphocytes, age, sex and tumor stage, the association of the EBV score with prognosis remained significant in the multivariate Cox regression model. Patients with high EBV scores have a 55% or 61% lower risk of progression than those with low scores using the tEBV (Figure 6E) or nEBV (Figure 6F) model, respectively.

## 4. Discussion

EBV+ gastric cancers represent a distinct subtype with favorable prognosis and high immune infiltration, making them ideal immunotherapy candidates [7]. Indeed, several studies report high overall response rates of EBV+ gastric cancer patients receiving immune checkpoint inhibitor therapy [9]. In this study, we developed a deep learning framework based on H&E slides to calculate an EBV score. Our model was proved to be accurate in both tumor and normal regions within each slide. Furthermore, we found our model could be partly interpreted by regional immune infiltration and could provide additional prognostic values beyond EBV status and immune infiltration. 

In recent years, deep learning has emerged as a powerful tool for medical image analysis. With the development of image processing techniques, deep learning methods are beginning to show increasing advantages over humans in various fields such as denoising, feature extraction and dimensionality reduction of medical images [30]. Previous studies have achieved high performance with deep learning methods for tumor diagnosis based on radiomics data [31]. Furthermore, many studies have successfully applied deep learning frameworks in predicting somatic mutations, pathway deficiencies and even viral infections using histological slides [14,15,16,32]. However, deep learning methods are mostly used as a black box. Most studies lack a clear interpretation of their deep learning methods. It is unclear what features deep learning models are capturing for prediction despite their high performance. In this study, we examined correlations between the model predictions and the infiltration of regional immune cells. There were positive correlations for the majority of slides even after stratifying by EBV abundance. Therefore, our model can be explained at least partly by regional immune infiltration within slides. Similarly, it is also likely that models in previous studies that successfully predicted somatic mutations as well as other molecular alterations captured information of regional immune infiltration for prediction. Indeed, the mutations of genes with high predictive accuracy such as TP53, STK11 and EGFR are often associated with high immune infiltration [33]. Additionally, the number of lymphocytes in each slide detected using deep learning methods was recently successfully used to predict the mutations of a number of genes, such as FGFR3 and TP53 [34]. 

Our model detected morphometric feature changes caused by EBV infection, including immune infiltration, rather than EBV genome. Interestingly, we found both tEBV and nEBV scores were more prognostic compared to EBV status and immune infiltrations. A possible reason is that the EBV model also capture other features such as immune cell subtypes in addition to local immune infiltrations. Due to the heterogeneous nature of immune cells in the tumor microenvironment, in addition to tumor-killing cells (e.g., CD8+ T cells), there are also immune cells (e.g., T regulatory cells) that can help tumors escape from immune surveillance. Therefore, considering cell subtypes may give our model higher prognostic values compared to overall immune infiltration.

Compared to traditional molecular techniques for detecting EBV, our method has the following advantages. (1) Our model is much faster than traditional techniques. In situ hybridization usually takes 2–3 days. After training, our model only requires a few minutes to tessellate a slide and make predictions. (2) Molecular techniques usually require good preservation of nucleic acids in slides, which can be degraded in tissue fixation, staining, etc. Our model directly works on H&E slides regardless of the preservation of nucleic acid, thereby expanding the range of samples available for testing. 

While we provided a promising method for detecting EBV infection status, there are some limitations to this study. First, the number of EBV+ samples in TCGA datasets is limited. Due to data availability, we have not validated our models in independent datasets. Further validation of the model’s performance in independent gastric cancer datasets will be needed. Second, TIL maps are binary-colored, with red indicating highly lymphocyte-infiltrated regions. If lymphocyte counts are taken into consideration, there should be a much higher correlation between immune infiltration and EBV score. 

## 5. Conclusions

In summary, we developed a deep learning-based algorithm to predict EBV status directly from H&E slides. Compared to traditional molecular techniques, our method is more efficient and economical. Our model is interpretable by regional infiltrating lymphocytes and can provide additional prognostic utilities. The results of this study may be extended in the future to develop more precise and interpretable models and to help predict patient responses to immunotherapy.

## Data Availability

H&E histology slides of TCGA gastric cancer samples used in this study are openly available in Genomic Data Commons through https://portal.gdc.cancer.gov/ (accessed on 15 March 2021). The TIL maps used in this study are openly available in The Cancer Imaging Archive (TCIA) at [doi.org/10.7937/K9/TCIA.2018.Y75F9W1].

## Supplementary Materials

The following are available online at https://www.mdpi.com/article/10.3390/cancers13236002/s1, Figure S1: Composition of the training and test sets, Figure S2: Model performance on the leave-out set, Figure S3: Association of HHV5 with immune infiltration, Figure S4: Enriched gene ontologies for high correlated genes, Figure S5: Local correlations between regional immune infiltration and EBV scores in samples with low EBV abundance. Supplementary material.docx: Description of supplementary figures.
