# Supplementary material for: Deep Learning Predicts EBV Status in Gastric Cancer Based on Spatial Patterns of Lymphocyte Infiltration
Source: Cancers (Basel). 2021 Nov 29;13(23):6002. doi: 10.3390/cancers13236002 (PMC8656870; doi:10.3390/cancers13236002)
Supplement: Supplementary file 1 [file cancers-13-06002-s001.zip › cancers-1442689-SI.pdf]

Supplementary material

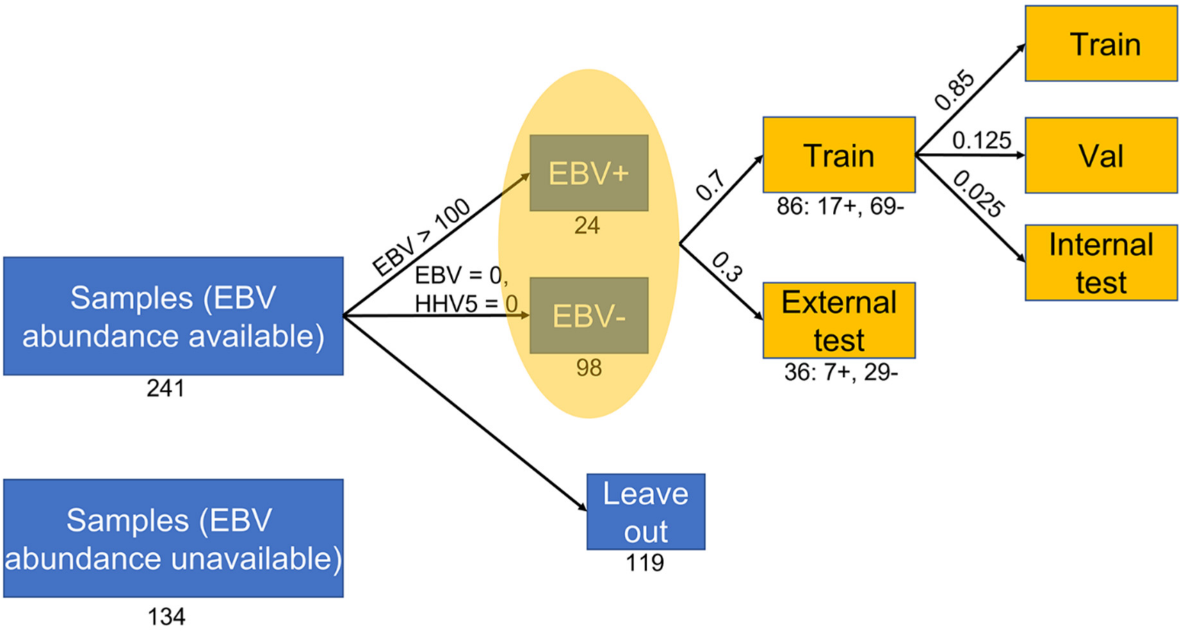

Figure S1. Composition of the training and test sets. Within 375 samples, 241 samples have EBV abundance information. Out of the 241 samples, 24 samples with EBV abundance above 100 RPHM were considered as EBV+, while 98 samples with zero EBV and HHV5 abundance as EBV-. 119 samples left out were considered as EBV status unclear. Training and test set were divided at sample levels at ratio 0.7:0.3. Training set was further divided into training, validation and internal test set on tile levels with ratio 0.85:0.125:0.025.

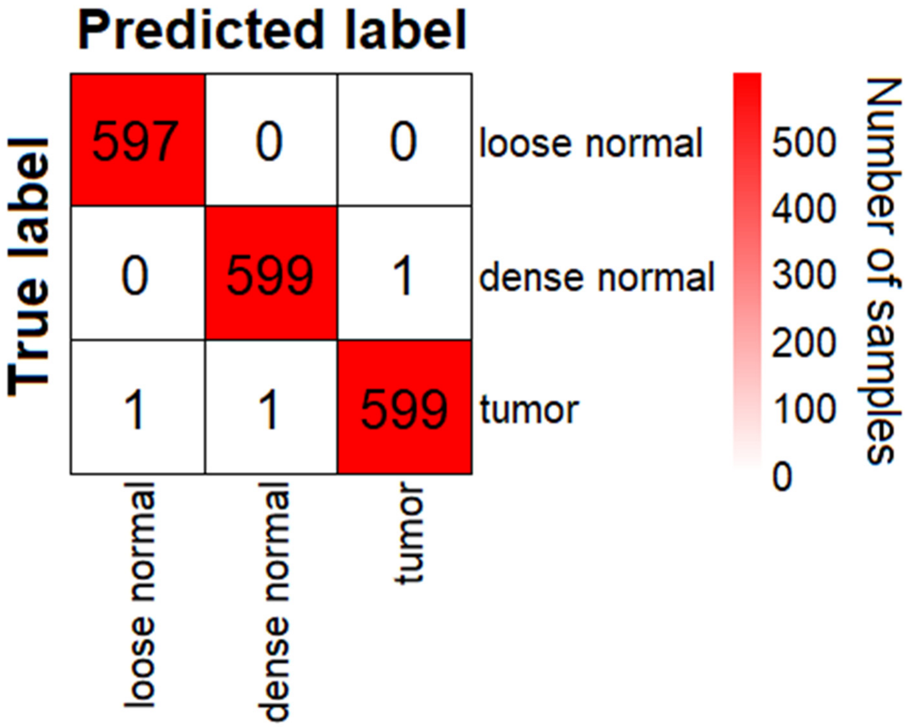

Figure S2. Model performance for classifying tumor and normal tiles. The confusion matrix for the tumor vs. normal model performance in test set were shown.

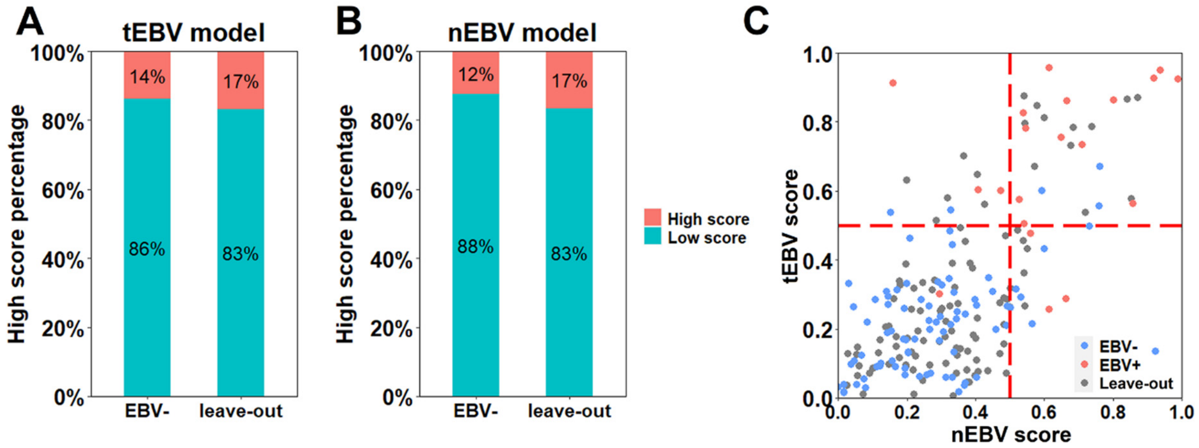

Figure S3. Model performance on the leave-out set. A-B: Both tEBV **(A)** and nEBV **(B)** models predict the majority of leave-out samples as EBV-. **C:** Prediction results of tEBV and nEBV models in the entire cohort.

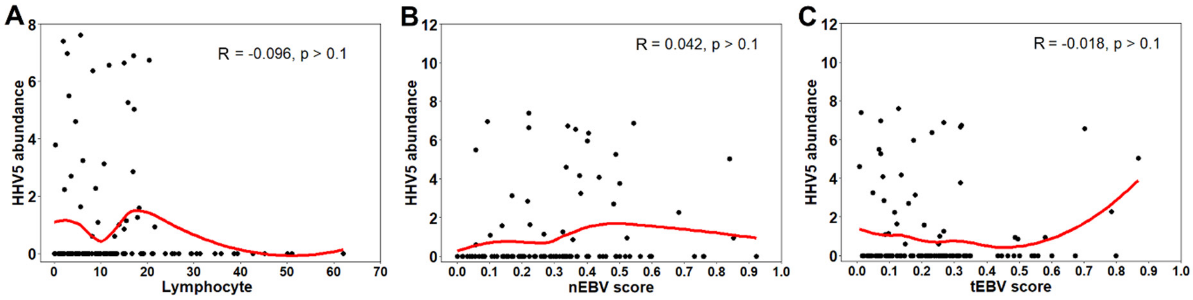

Figure S4. Association of HHV5 with immune infiltration. A-C: HHV5 does not correlate with immune infiltration **(A)** nor EBV scores from tEBV **(B)** or nEBV **(C)** model.

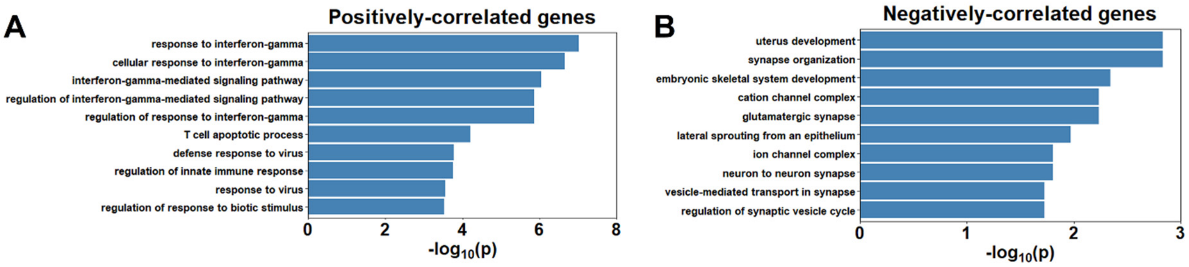

Figure S5. Enriched gene ontologies for high correlated genes. A-B: Enriched gene ontologies for positively **(A)** or negatively **(B)** correlated genes with tEBV, nEBV scores and EBV abundance.

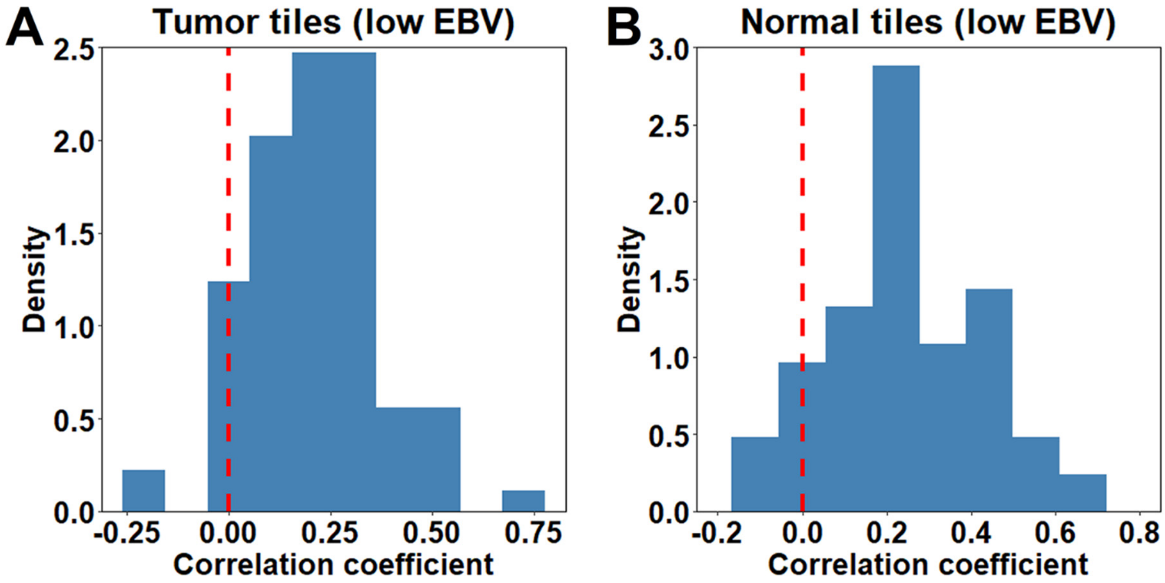

Figure S6. Local correlations between regional immune infiltration and EBV scores in samples with low EBV abundance. A-B: Majority slides with low EBV abundance (0~100 RPHM) show positive correlations of regional immune infiltrations with tEBV **(A)** or nEBV **(B)** scores.
