# Supplementary figures and images for: Deep Learning Predicts EBV Status in Gastric Cancer Based on Spatial Patterns of Lymphocyte Infiltration
Source: Cancers (Basel). 2021 Nov 29;13(23):6002. doi: 10.3390/cancers13236002 (PMC8656870; doi:10.3390/cancers13236002)

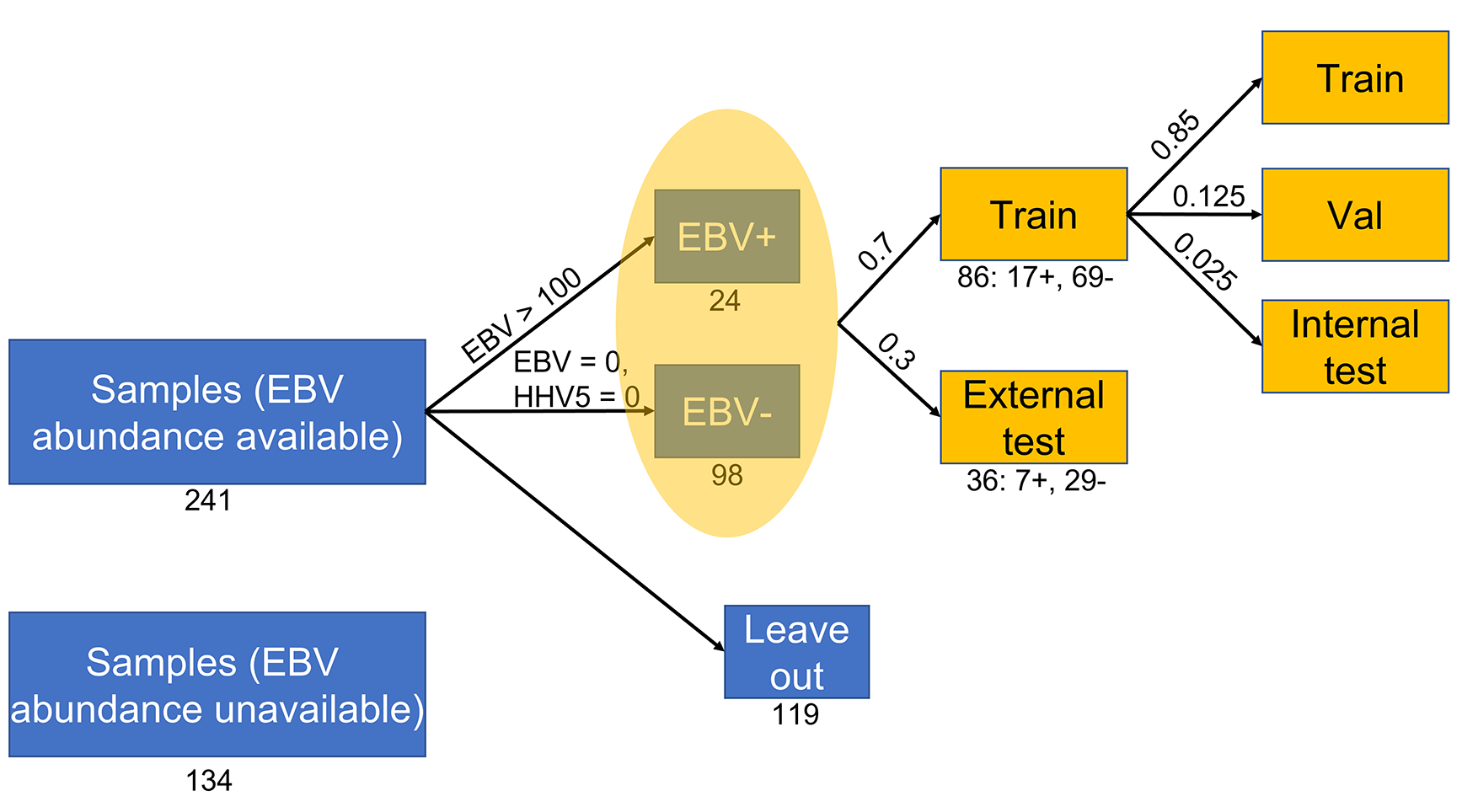

Supplement: Supplementary file 1 [file cancers-13-06002-s001.zip › Figure S1.png]

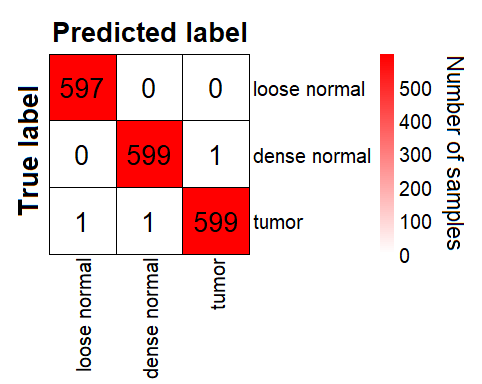

Supplement: Supplementary file 1 [file cancers-13-06002-s001.zip › Figure S2.png]

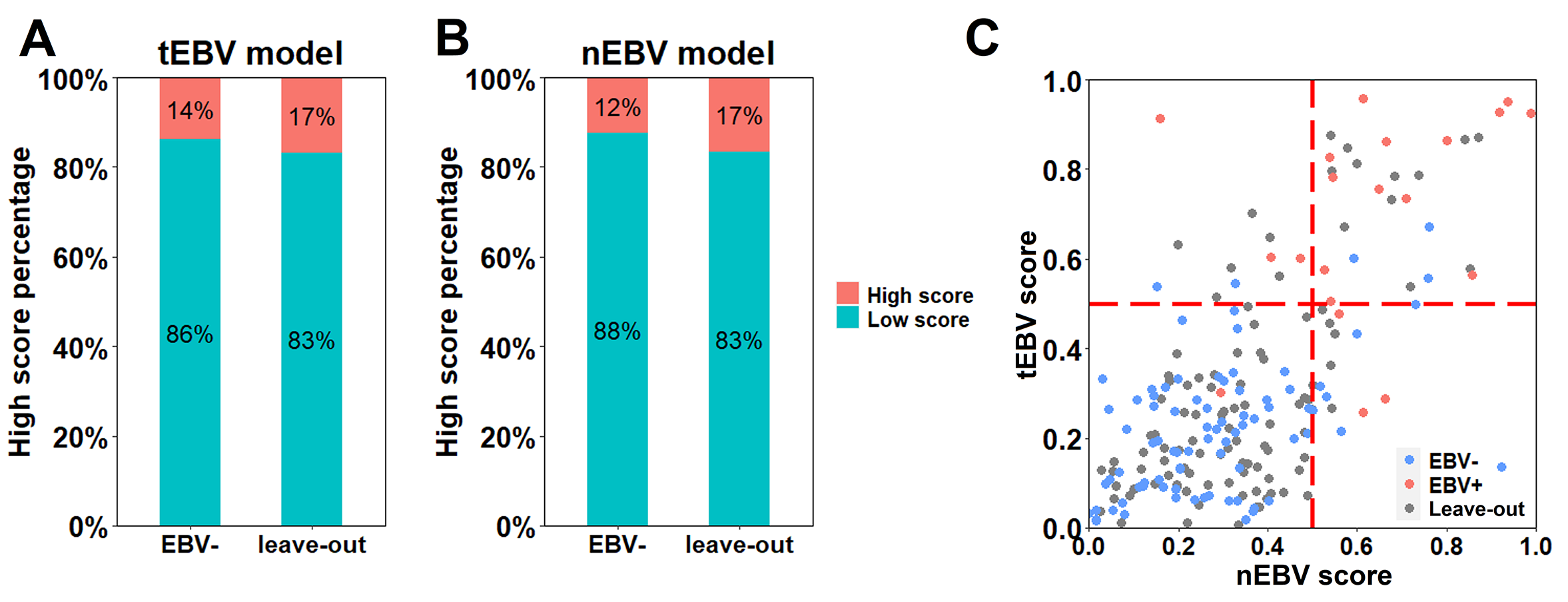

Supplement: Supplementary file 1 [file cancers-13-06002-s001.zip › Figure S3.png]

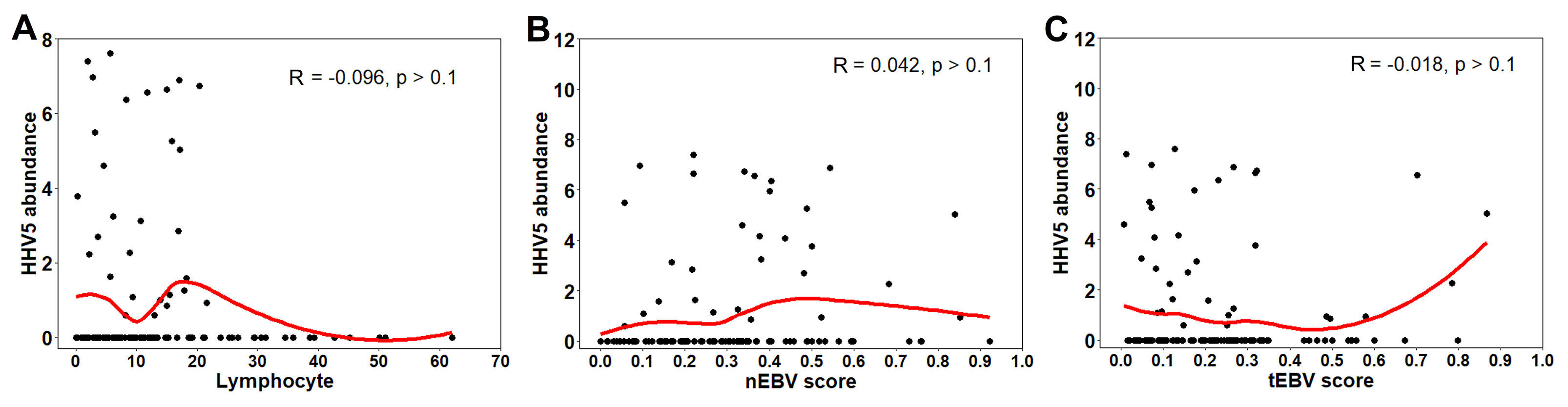

Supplement: Supplementary file 1 [file cancers-13-06002-s001.zip › Figure S4.png]

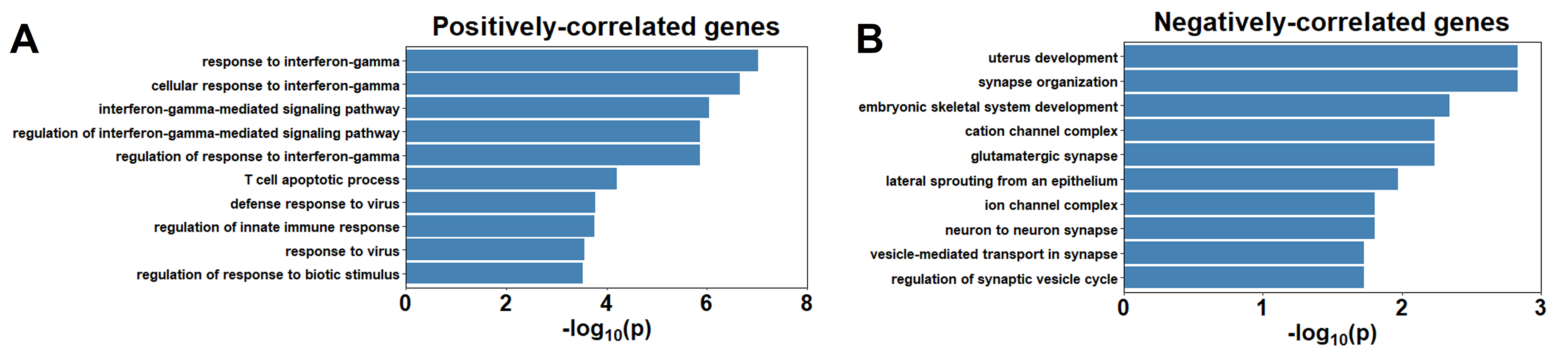

Supplement: Supplementary file 1 [file cancers-13-06002-s001.zip › Figure S5.png]

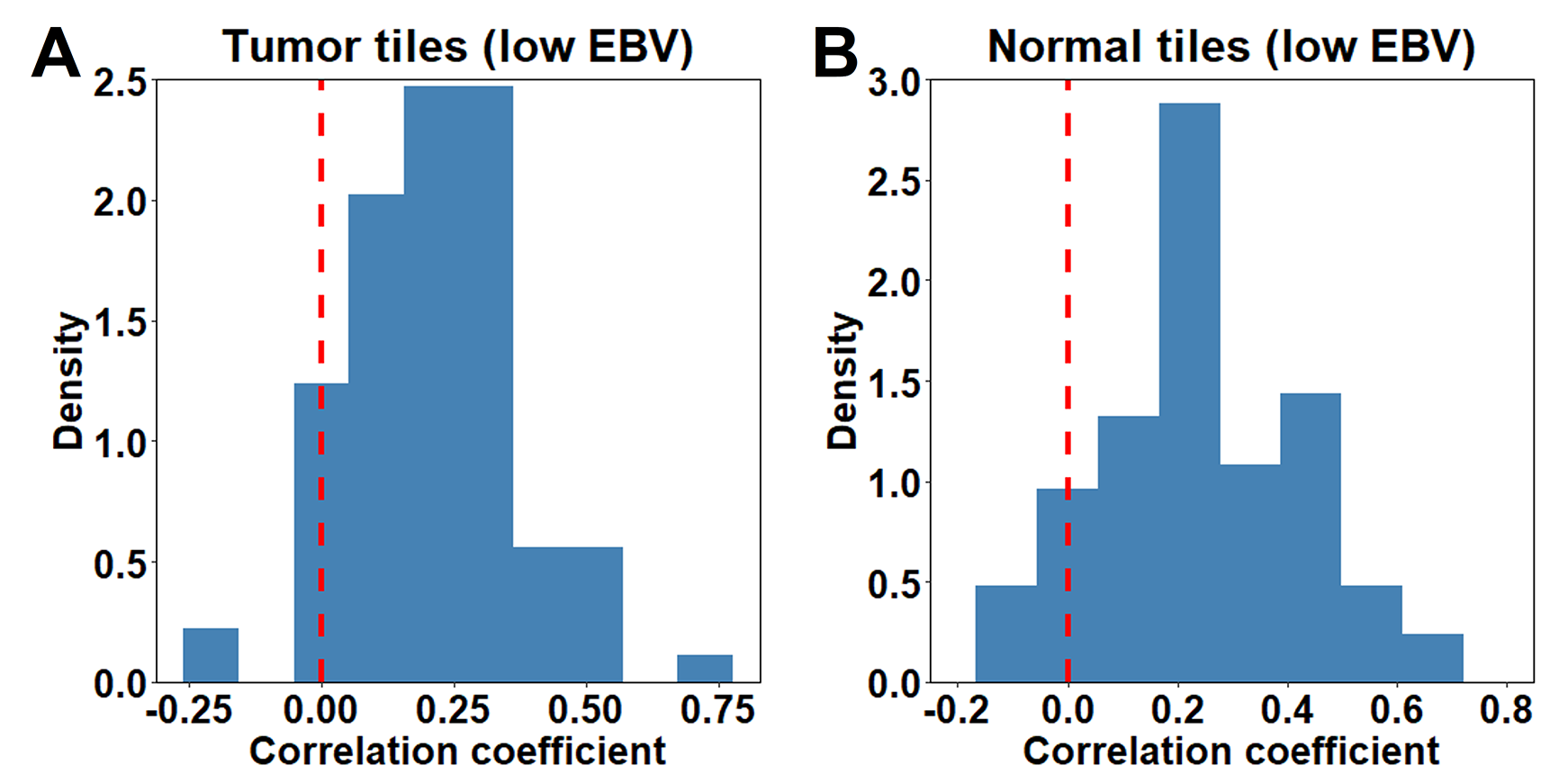

Supplement: Supplementary file 1 [file cancers-13-06002-s001.zip › Figure S6.png]
